# Brain Imaging, Forward Inference, and Theories of Reasoning

**DOI:** 10.3389/fnhum.2014.01056

**Published:** 2015-01-09

**Authors:** Evan Heit

**Affiliations:** ^1^School of Social Sciences, Humanities and Arts, University of California Merced, Merced, CA, USA

**Keywords:** reasoning, neuroimaging, deduction, induction, forward inference

## Abstract

This review focuses on the issue of how neuroimaging studies address theoretical accounts of reasoning, through the lens of the method of forward inference (Henson, 2005, 2006). After theories of deductive and inductive reasoning are briefly presented, the method of forward inference for distinguishing between psychological theories based on brain imaging evidence is critically reviewed. Brain imaging studies of reasoning, comparing deductive and inductive arguments, comparing meaningful versus non-meaningful material, investigating hemispheric localization, and comparing conditional and relational arguments, are assessed in light of the method of forward inference. Finally, conclusions are drawn with regard to future research opportunities.

How can neuroimaging techniques help address theoretical questions in reasoning research? To be more specific, how can techniques such as functional magnetic resonance imaging (fMRI) help researchers distinguish between psychological theories of reasoning? There have been thousands of behavioral experiments on reasoning, and the field as a whole has several competing theories without a consensus of which one best account for the behavioral data. Potentially, new evidence on patterns of brain activity during reasoning tasks could help resolve these long-standing debates.

This article will first briefly outline several psychological theories of deductive and inductive reasoning. Next, a particular method (forward inference; Henson, 2005, 2006) for using neuroimaging data to test predictions from psychological theories will be critically discussed. Then, example neuroimaging studies of deductive and inductive reasoning will be reviewed, through the lens of the method of forward inference. By no means is forward inference the only possible means to advance psychological theory in the context of neuroimaging. This exercise will provide some perspective both on neuroimaging studies of reasoning and on the method of forward inference.

## Theories of Reasoning

Researchers have studied reasoning on both problems of deduction and problems of induction. Problems of deduction require drawing a valid, logical conclusion that must follow based on a set of given premises. In contrast, problems of induction require drawing probabilistic conclusions from given information as well as other relevant knowledge (Heit, 2007; Hayes et al., 2010). One open question in reasoning research is whether deduction and induction simply refer to two different kinds of reasoning problems – in terms of the structure and/or content of the problems themselves – or if there are truly two different kinds of reasoning, deductive reasoning and inductive reasoning, with different cognitive processes (or different mixtures of cognitive processes) involved (Rotello and Heit, 2009; Heit and Rotello, 2010; Heit et al., 2012).

According to dual-process accounts [e.g., Kahneman (2011), Evans and Stanovich (2013)], there are two kinds of underlying mechanisms, heuristic processing and analytic processing. Both induction and deduction could be influenced by these two processes, but in different mixtures (Rotello and Heit, 2009; Heit and Rotello, 2010; Heit et al., 2012). Under this mixture account, induction judgments could be particularly influenced by heuristic processes that tap into associations and knowledge that do not necessarily make an argument logically valid. In contrast, deduction judgments could be more heavily influenced by slower analytic processes that encompass more deliberative, and typically more accurate, reasoning. However, for present purposes, the crucial point is that there are two processes, not the details of any possible mixture.

In comparison, single-process accounts explain reasoning in terms of a common set of mechanisms across multiple forms of reasoning, although typically these theories focus more on either deduction or induction. Mental model theory (Johnson-Laird, 1994) asserts that a reasoner assesses an argument by constructing a visuospatial model of the premises then looking for counterexamples. Although this theory is typically applied to problems of deduction, it has also been applied to problems of induction. Bayesian accounts of reasoning address performance on problems of deduction in terms of making probabilistic judgments (Oaksford and Chater, 2007); hence, they are inductive in nature. Indeed, related models of inductive reasoning are also Bayesian in nature (Heit, 1998; Tenenbaum and Griffiths, 2001). Additionally, there are some models of inductive reasoning (Osherson et al., 1990; Sloman, 1993) that focus on problems of induction but can address performance on some problems of deduction as well. Finally, mental logic theory (Rips, 1994; Braine and O’Brien, 1998) has focused on deduction, asserting that people reason on problems of deduction by carrying out syntactic operations using a system of logical rules.

## Drawing Theoretical Inferences

Although there has been skepticism about drawing inferences about psychological theories from neuroimaging data [e.g., Coltheart (2006), Harley (2004), Uttal (2011), Van Orden and Paap (1997)]. Henson (2005, 2006) has outlined a rationale for doing so, adopting standard notions from experimental psychology on employing behavioral data. Henson (2006) referred to this process as “forward inference,” namely, “the use of qualitatively different patterns of activity over the brain to distinguish between competing cognitive theories.” The key idea is that if theory 1 predicts that the same cognitive processes underlie two different experimental tasks, and theory 2 predicts that the tasks differ in terms of at least one cognitive process, then theory 2 will be supported when patterns of brain activity differ between the two tasks. This inference depends on the assumption that there is at least some systematic mapping between cognitive processes and brain regions, namely, the weak assumption that within the experimental comparison of interest, the same cognitive process is not supported by different brain regions.

Forward inference itself has some limitations, such as its asymmetrical nature, that is, theory 1 can be supported by null results, whereas theory 2 could potentially be supported numerous differences. Also, as Henson (2006) noted, forward inferences are theory-dependent, namely, theories 1 and 2 may both be incorrect, and some alternative account such as theory 3 may be correct. If that alternative is not considered by the researcher, then forward inferences based on theories 1 and 2 will be misleading. Another pitfall is that there can be other reasons for differences in localization, namely, if two experimental tasks differ in patterns of brain activity, the reason may not be differences in cognitive processes but differences in rate of responding “yes” [Nosofsky et al. (2012); for a related argument, involving task complexity, see Johnson (1993)]. Going beyond the issue of which regions are activated is the matter of how these activations are causally related to each other [e.g., Chiong et al. (2013)]. In general, as Monti and Osherson (2012) point out, reasoning “should be regarded as a collection of processes and representations” [cf., Anderson (1978)], hence observed differences may correspond not to processing differences but differences in the content being processed.

A more fundamental problem for forward inference is that the theories of interest simply may not make predictions about brain activity. In Marr’s (1982) terms, the theories may be at the algorithmic or computational level of description, without strong connections to the implementation level. Henson (2005) was optimistic, however, that brain imaging could either directly address the algorithmic level of processing or do so indirectly, by illuminating the implementation level which itself would constrain the algorithmic level.

A companion article to Henson (2006), by Poldrack (2006), described “reverse inference,” by which the presence of a particular cognitive process is inferred from a pattern of brain activity [see Del Pinal and Nathan (2013), for a critical review]. Poldrack noted that a researcher’s confidence in a reverse inference can be explained in terms of Bayes’s Theorem, with the conditional probability that the cognitive process is engaged when a particular brain region is activated depending, in part, on the prior likelihood that cognitive process appears in the experimental context. Put another way, if the cognitive process is implausible in absolute terms, then the researcher should not be greatly confident that it is tied to any particular brain region. This point echoes the situation in forward inference that if two theories being compared are both incorrect, then imaging results could only give misleading support for one over the other. The conditional probability also depends on the selectivity of the brain region. For example, if the brain region is so large that it is activated by many cognitive processes, then it will be difficult to infer the engagement of any one process when the region is activated.

Although reverse inference is not used to directly compare theories, it is a part of the scientific process that could be used to develop theories. Moreover, Poldrack’s (2006) Bayesian formulation of reverse inference inspires a Bayesian generalization of forward inference, as shown in Eq. 1.

(1) $$\begin{array}{llll} & P\left(\mathrm{theor}y_{1}|\mathrm{results}\right)\; & & \;\\ & \;=\frac{P\left(\mathrm{results}|\text{theor}y_{1}\right)P\left(\mathrm{theor}y_{1}\right)}{\begin{array}{c} P\left(\mathrm{results}|\text{theor}y_{1}\right)P\left(\mathrm{theor}y_{1}\right)+P\left(\mathrm{results}|\text{theor}y_{2}\right)\times \\ \;P\left(\mathrm{theor}y_{2}\right)+\ldots +P\left(\mathrm{results}|\text{theor}y_{n}\right)P\left(\mathrm{theor}y_{n}\right) \end{array}} \end{array}$$

Here, the conditional probability that theory 1 is correct after observing a set of neuroimaging results depends on the conditional probability of the results under that theory, as well as the prior likelihood of the theory. This probability must be normalized in terms of the likelihood of other, competing theories. Forward inference is a special case with two theories and the observed results being either the same pattern of brain activity across two experimental tasks or different patterns of brain activity.

## Predictions about Brain Activity

Next, several examples of neuroimaging studies of reasoning, aiming to address theoretical views, will be reviewed in the light of the method of forward inference.

### Deduction versus induction

At least one of the contrasts made in imaging research on reasoning is a good example of forward inference. Several studies (Goel et al., 1997; Osherson et al., 1998; Parsons and Osherson, 2001; Goel and Dolan, 2004) have compared deductive and inductive reasoning tasks. One class of theories (including mental model theory and Bayesian accounts) has suggested that deduction and induction are performed by a common set of processes. Another class of theories (dual-process theories) has suggested that there are two types of underlying mechanisms of reasoning, heuristic and analytic processing, which would contribute differentially to deduction and induction. To the extent that different patterns of brain activity are observed for deduction versus induction tasks, holding everything else equal between experimental conditions, by forward inference, dual-process accounts will be supported over single-process alternatives. (Note that three of these studies, all but Goel and Dolan, 2004, used exactly the same materials for the two conditions, but simply asked a deduction question or an induction question.) Indeed, these four studies all found somewhat different patterns of brain activation for deduction versus induction. Three of these studies (Goel et al., 1997; Osherson et al., 1998; Goel and Dolan, 2004) found increased activation for induction, relative to deduction, in left frontal cortex, although in somewhat different regions at a finer level. Although it would be valuable to have an understanding of why the regions differ between studies, which is not crucial for the method of forward inference.

Overall, these results do make a good case for dual-process theories over single-process theories, notwithstanding the limitations of forward inference described above. To accommodate, these results would require single-process theories to assume somewhat different processes for deduction versus induction, e.g., to become more like dual-process theories. In a related line of work Houdé et al. (2000, 2001) compared brain activity before and after a training session aimed at improving logical reasoning, rather than comparing reasoning under two sets of instructions. In terms of the method of forward inference, the qualitatively different patterns of activity pre- versus post-training would be a challenge for single-process accounts, without assuming that deduction before and after training engages different processes.

### Meaningful versus non-meaningful material

Another contrast is a slightly less clear example of forward inference. Several studies have varied the content of arguments while otherwise keeping the task the same, e.g., abstract versus concrete materials (Goel et al., 2000; Goel and Dolan, 2001), materials that agree, disagree, or are neutral with respect to prior knowledge (Goel and Dolan, 2003), and visual versus spatial relations such as “fatter than” versus “is a descendant of” (Knauff et al., 2003). To apply forward inference, what is needed is one theory that predicts the same cognitive processes between conditions, and another theory that predicts different cognitive processes between conditions. With regard to the abstract/concrete and prior knowledge studies, the results were greater bilateral parietal activation for abstract or neutral content, and in two of the studies, greater left temporal activation for concrete or knowledge-related materials. With regard to the study on visual versus spatial relations, the finding was that visual problems led to enhanced activity in visual association cortex. Although these differences in brain activity would be consistent with dual-process accounts assuming that somewhat different mechanisms are employed depending on content, the problem is that even single-process accounts would need to make some assumptions to explain how content affects reasoning. So it is unclear that single-process accounts are ruled out [cf., Keren (2013)]. From the perspective of forward inference, the problem is the lack of well-defined theories making sharply different predictions.

### Left versus right hemisphere

A frequent prediction addressed in brain imaging research on reasoning is whether the left or right hemisphere is activated. It is tempting to link mental logic theory, having a propositional nature, with left hemisphere activation and mental model theory, having a visuospatial nature, with right hemisphere activation. Therefore, by looking at which hemisphere is predominantly activated during a reasoning task, one might see which theory has greater support. With regard to mental model theory, the origin of this prediction appears to be Johnson-Laird (1994), and it has been tested in many studies (Goel et al., 1997, 1998, 2000; Parsons and Osherson, 2001; Knauff et al., 2002, 2003; Noveck et al., 2004; Monti et al., 2007, 2009). Although reasoning tasks are typically associated with left hemisphere activation, the results have actually been mixed (Goel, 2007), with many studies showing activation in both hemispheres.

Of greater concern is not the result but the soundness of the hemispheric prediction. An inference of the form “if theory X is correct then brain region Y will be activated” is neither forward inference nor reverse inference. Indeed, no proponent of either theory of reasoning would likely abandon their beliefs based on tests of these predictions. Noveck et al. (2004) suggested that no proponent of mental logic theory has even made predictions about brain regions. Moreover, the predictions about brain regions are not unique, e.g., alternative predictions can also be made for mental model theory, such as parietal activation (Knauff et al., 2003) or activation in the anterior prefrontal cortex (Fangmeier et al., 2006). Knauff et al. even suggested that left hemisphere activation may be consistent with mental model theory, because comprehension of arguments will recruit linguistic areas of the brain.

A final problem with the hemispheric prediction is that it sets up a comparison between two theories that are not the only possibilities. In terms of Eq. 1, other theories need to be considered. For example, the studies reviewed here did not consider Bayesian accounts of deduction (Oaksford and Chater, 2007), yet these accounts have amassed a growing set of successes in the domain of reasoning.

### Conditional versus relational arguments

Other neuroimaging studies (Knauff et al., 2002; Prado et al., 2010) have compared reasoning about two types of deduction problems, conditional (if-then) arguments and relational arguments (e.g., regarding relative spatial position). The Knauff et al. study was largely concerned with hemispheric predictions comparing mental model and mental logic theory. There were some differences in activation when comparing the two argument types; however, these differences were bilateral and not interpreted strongly. Prado et al. were more directly interested in comparing the two argument types, and indeed observed that the left inferior frontal gyrus is activated more for conditional arguments and the right temporo-parieto-occipital region is activated more for spatial arguments. These results were interpreted as evidence against “unitary” accounts of deduction and evidence for “fractionated” accounts of deduction. To the extent that unitary views predict that the same cognitive processes are used for the two tasks, and fractionated views predict that different processes are used, this is a good example of forward inference. Prado et al. took a particularly nuanced approach, pointing out that although mental model and mental logic theory can be treated as unitary accounts, it is possible to imagine “hybrid” versions predicting somewhat different cognitive processes depending on argument type. Hence, the results are useful in ruling out basic versions of single-process accounts of reasoning. However, the problem, in terms of forward inference and Eq. 1, is that multiple theories of the fractionated type, which is multiple theories that predict that different processes will underlie different problems, are still possible. So there is negative evidence against some theories but the distinctive, positive evidence for other theories is less clear.

For further discussion, including a meta-analysis of brain imaging studies across argument types and presentation modalities, see Prado et al. (2011) for an extended argument that deductive reasoning is better described in terms of multiple systems than a single mechanism.

## Conclusion

Just as researchers spell out all of the methodological details of brain imaging studies, it is valuable when researchers spell out the details of their own reasoning, e.g., list alternative theories, give sources for predictions, examine alternative predictions, and explain the rationale of testing predictions. The method of forward inference is one such rationale, although as discussed, it is not without its own limitations. This review of brain imaging studies of reasoning has shown that some comparisons, namely, deduction versus induction and conditional arguments versus relational arguments, have made profitable use of forward inference. The possible theoretical contributions of other studies reviewed here appears to lie outside of forward inference, likely reflecting limitations of forward inference as well as cases where the studies need a more fully spelled-out rationale for making theoretical comparisons.

Looking to the future, another approach with great promise is to combine neuroimaging with mathematical modeling, to test well-specified psychological theories. Indeed, some methods of combining neuroimaging and modeling can be seen as extensions or generalizations of the method of forward inference, providing alternative methods for distinguishing between psychological processing accounts using neuroimaging data. For example, rather than comparing a single-process account to a dual-process account, McClure et al. (2007) implemented a mixture model comprising two processes, with the aim of linking model parameters to localized brain activity. Staresina et al. (2013) used the method of state-trace analysis to look for non-monotonic patterns of brain activity across experimental conditions that would rule out single-process accounts. Mack et al. (2013) compared patterns of brain activation to latent model representations for competing psychological models, assessing the match between brain activity and model predictions across multiple experimental manipulations. Finally, Rotello and Heit (2014) reinterpreted brain imaging studies of conflicts between prior beliefs and deductive reasoning, seeming to show multiple reasoning processes, using an algebraic analysis based on signal detection theory.

## Conflict of Interest Statement

The author declares that the research was conducted in the absence of any commercial or financial relationships that could be construed as a potential conflict of interest.
